# Radiologist Checklist for Selecting Patients to Undergo PIPAC (Pressurized IntraPeritoneal Aerosol Chemotherapy)

**DOI:** 10.3390/life11090941

**Published:** 2021-09-09

**Authors:** Elena Rodolfino, Margo’ Di Marco, Alessia Ilot, Roberto Iezzi, Benedetta Gui, Giacomo Avesani, Camilla Panico, Antonia Strippoli, Andrea Di Giorgio, Fabio Pacelli, Riccardo Manfredi

**Affiliations:** 1 Department of Diagnostic Imaging, Oncological Radiotherapy and Hematology, Fondazione Policlinico Universitario “A. Gemelli” IRCCS, 00168 Rome, Italy; elena.rodolfino@policlinicogemelli.it (E.R.); benedetta.gui@policlinicogemelli.it (B.G.); giacomo.avesani@policlinicogemelli.it (G.A.); camilla.panico@guest.policlinicogemelli.it (C.P.); riccardo.manfredi@unicatt.it (R.M.); 2 Section of Radiology, Department of Radiological and Hematological Scienses, Università Cattolica del Sacro Cuore, 00168 Rome, Italy; margo.dimarco01@icatt.it (M.D.M.); alessia.ilot01@icatt.it (A.I.); 3 Comprehensive Cancer Center, Fondazione Policlinico Universitario “A. Gemelli” IRCCS, 00168 Rome, Italy; antonia.strippoli@policlinicogemelli.it; 4 Surgical Unit of Peritoneum and Retroperitoneum, Fondazione Policlinico Universitario “A. Gemelli” IRCCS, 00168 Rome, Italy; andrea.digiorgio@policlinicogemelli.it (A.D.G.); Fabio.pacelli@policlinicogemelli.it (F.P.)

**Keywords:** peritoneal carcinomatosis, PIPAC, pressurized intraperitoneal aerosol chemotherapy, PCI

## Abstract

Peritoneal carcinomatosis frequently occurs in advanced gastrointestinal and gynecological cancers. As factors such as poor drug uptake and distribution cause chemotherapy to be less effective, alternative therapies have been explored. Introduced in 2013, PIPAC (pressurized intraperitoneal aerosol chemotherapy) uses aerosolized chemotherapeutics sprayed into the patient’s peritoneal cavity using a laparoscopic approach. Despite the literature showing encouraging data regarding the tolerability and efficacy of PIPAC, there is a lack of articles on the role that imaging plays in selecting patients suitable for PIPAC. The aim of this study is to combine literature-based evidence and clinical experience to provide information able to support training radiologists, as well as experienced radiologists interested in innovative therapies.

## 1. Introduction

In advanced stages of gastrointestinal and gynecological cancers, peritoneal carcinomatosis frequently occurs due to disease progression and poor prognosis.

Due to pharmacokinetic limitations, poor peritoneal drug uptake, and impaired local drug distribution, systemic chemotherapy is not as effective in peritoneal metastases as in sites like the liver or lung.

Consequently, new alternative therapies have been proposed with the ideal aim of safely delivering a highly selective dose of chemotherapy drug to peritoneal carcinomatosis (PC) lesions, using a minimally invasive platform. The recipients of these alternative approaches are patients with recurrent cancer and metachronous disease who do not benefit from palliative systemic chemotherapy or cannot undergo systemic chemotherapy.

In 2013, Prof. M. A. Reymond proposed PIPAC (pressurized intraperitoneal aerosol chemotherapy), a new intraperitoneal treatment approach. This procedure comprises of the spraying of aerosolized chemotherapeutics directly into the patient’s abdominal peritoneal cavity during abdominal laparoscopy. First reports seem to show that PIPAC is well-tolerated, allowing increased locoregional delivery and tissue penetration of chemotherapy by hyper pressure, reducing the concentration of drugs used compared with those intravenously [1].

This new therapy minimizes systemic side effects, improving patient quality of life and extending survival rates [1,2]. According to the literature, it is effective in 50–88% of patients with advanced peritoneal metastasis, refractory to standard treatment [3,4]. The real role and timing of PIPAC in the treatment of peritoneal carcinomatosis has yet to be investigated, particularly in relation to the possibility of being combined with additional palliative intravenous chemotherapy without any significant change in terms of quality of life [2,5,6].

However, to date, there are no published articles on the role played by imaging in selecting patients suitable for PIPAC.

The aim of this article is to integrate evidence reported in the literature and perceptions based on clinical experience. In addition, our purpose will be to make information easy to access, for assisting not only residents and fellows who are training in abdominal radiology, but also experienced radiologists who want to know more about this new experimental surgical approach. In detail, the role of the radiologist is essential in multidisciplinary teams for recognizing disease-related criteria that may make PIPAC an indicated or contraindicated approach.

## 2. Material and Methods

A systematic review was carried out and reported according to Preferred Reporting Items for Systematic Review and Metanalysis (PRISMA) statement guidelines, using a combination of medical subject headings (MeSH), terms, and keywords including “PIPAC”, “Pressurized IntraPeritoneal Aerosol Chemotherapy”, “peritoneal cytoreduction”, “peritoneal carcinomatosis index”, “Computed Tomography”, “CT”, “Magnetic Resonance”, and “MRI”. The Medline search query was: ((peritoneal carcinomatosis index) OR (pipac)) OR (Pressurized IntraPeritoneal Aerosol Chemotherapy)) OR (peritoneal cytoreduction) AND ((Computed Tomography) OR (CT) OR (Magnetic Resonance) OR (MRI)). All full-text articles written in English were identified and reviewed. The article search was completed manually by screening references from relevant papers and using the snowball search technique.

## 3. Study Selection and Data Extraction

After removing duplicates, single retrieved citations were screened, based on reading the title and abstract. We extracted potentially relevant abstracts, full-text articles, and those who met the inclusion criteria were considered for final analysis. Two researchers (MDM, AI) performed citation screening independently; uncertainties about any inclusions in the review were resolved by two expert radiologists (ER, RI).

Articles that met the following eligibility criteria were selected for the final analysis: (a) studies on the use of diagnostic imaging in patients with advanced peritoneal carcinomatosis; (b) studies on the assessment of peritoneal carcinomatosis; (c) clinical studies on the use of PIPAC in advanced peritoneal carcinomatosis; (d) clinical studies on the feasibility of PIPAC in patients with advanced peritoneal carcinomatosis; (e) studies published in English between 2006 and 2021 (time restriction). Conference papers, duplicate publications, surveys, letters, editorials, book chapters, reviews, and abstracts were excluded.

## 4. Results

**Figure 1:**
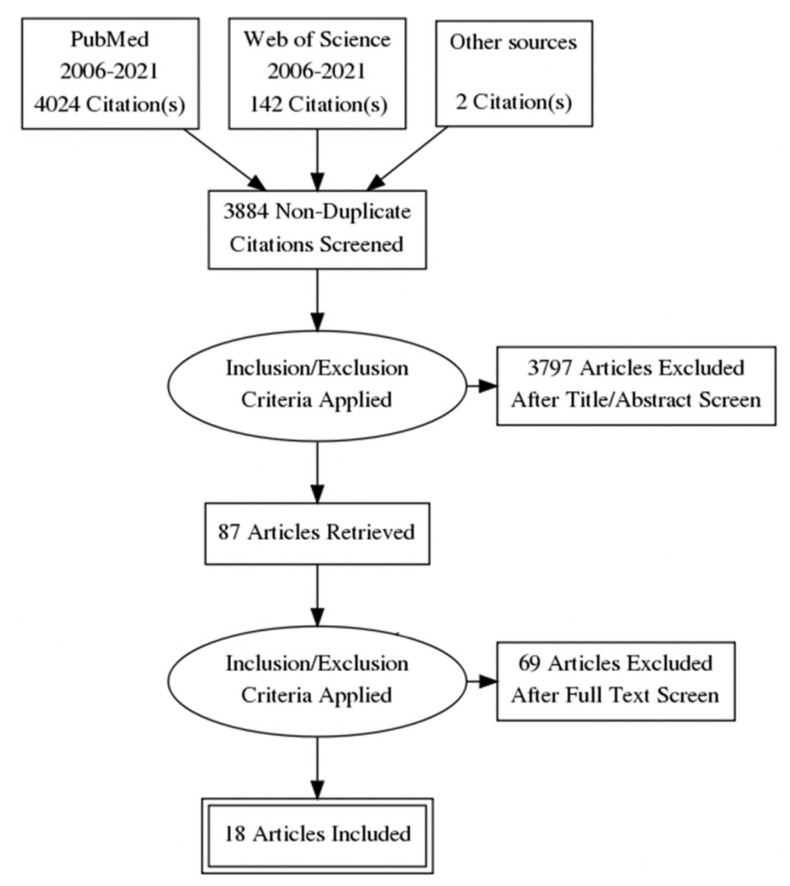
Flowchart of the study selection process.

The literature search strategy resulted in 3884 single citations. 87 articles were identified for full-text evaluation; out of these, 69 were excluded, with a final inclusion of a total of 18 articles. A flowchart of the study selection process is described in Figure 1.

All articles included were evaluated and discussed by the Peritoneum Board at our institute, composed of multidisciplinary experts, in order to integrate evidence reported in the literature and perceptions based on our clinical experience. Imaging modality, patient selection for PIPAC based on clinical and radiological patterns, quantitative and qualitative assessment of peritoneal carcinomatosis, extraperitoneal disease detection, and relative contraindications to PIPAC or laparoscopic approach were discussed.

The multidisciplinary expert team also defined a checklist based on these data and on the multidisciplinary experience of our center (Appendix A).

## 5. Imaging Modality

Characteristics of computed tomography (CT) such as wide availability, robust techniques and protocols, good resolution, and rapid acquisition times allow detection of both peritoneal and extra-peritoneal disease, making this imaging technique the reference for detection of peritoneal disease.

Commonly, a CT scan is performed by using a multi-detector helical scan before and after IV administration of contrast media, using double-phase acquisition (portal and delayed contrast-enhanced phase). In detail, in some small implants, a contrast resolution can be increased through a delayed phase acquired from 5 to 10 min after contrast injection. The use of an oral contrast medium (a negative contrast medium or a water density contrast) is recommended for optimal visualization of the bowel wall, serosa, and peritoneum [7].

In order to detect peritoneal disease and check the common peritoneal site of pathological involvement and distinct patterns of appearance, axial and other multi-planar reformatted (MPR) images can be useful.

Sensitivity and specificity for detecting PC are 83% (95% CI: 79–86%) and 86% (95% CI: 82–89%), respectively [8].

Nonetheless, the accuracy of a CT scan depends on lesion size, and results are less representative in small peritoneal implants. In detail, only 28% of small peritoneal nodules or masses less than 0.5 cm were detected, while 72% of moderate-size nodules of 0.5 to 5.0 cm were recognized, and 90% of gross nodules greater than 5 cm [9].

To balance the limits of CT scanning in detecting potentially missed small tumors, there is magnetic resonance imaging (MRI), which has superior contrast resolution.

MRI provides a powerful tool for preoperative evaluation of PC by combining different contrast mechanisms, including a dedicated MRI protocol that, besides diffusion-weighted imaging (DWI) and post-contrast series, involves proper bowel preparation—combining antispasmodics with negative oral contrast fluid to suppress signals from bowel contents [10].

## 6. Patient Selection for PIPAC

Patients are chosen for PIPAC based on clinical and radiological patterns; in particular, PIPAC is recommended for patients whose cancer has been stabilized by systemic chemotherapy but is not resectable due to extensive involvement, and who are not eligible for HIPEC and/or have chosen to stop IV chemotherapy, as well as patients who have developed resistance to systemic intravenous chemotherapy, or during first line treatment to reinforce IV chemotherapy in order to make an extensive peritoneal disease resectable [11].

Imaging is crucial in finding patients who are not eligible for CRS/HIPEC (cytoreductive surgery and hyperthermic intraperitoneal chemotherapy) but can be treated with PIPAC therapy. It is particularly helpful in obtaining a detailed evaluation of peritoneal carcinomatosis in terms of disease volume and duration before surgery, as well as to measure the radiological PCI score. It also allows precise qualitative detection of diseases that may complicate surgery or prevent optimal debulking, detects extraperitoneal metastases in anatomic sites inaccessible via laparoscopy, and distinguishes technological contraindications to PIPAC or laparoscopic approaches.

## 7. Quantitative Assessment of Peritoneal Carcinomatosis

### PCI Score

The Peritoneal Cancer Index (PCI) is a scoring method used to determine the extent of PC. According to P. H. Sugarbaker, severity of the disease is scored on a scale with a maximum value of 39 by distinguishing the abdomen into 13 distinct areas. The larger lesion in each of the 13 anatomical locations in the peritoneal cavity is assigned a score ranging from 1 to 3 based on its size: 0.5 cm, 0.5–5 cm, or >5 cm, respectively [12]. The PCI has been shown to predict the surgical team’s ability to conduct a full cytoreduction and is considered the best predictor of long-term survival currently available.

CT has been shown to often underestimate intraoperative PCI, while magnetic resonance imaging (MRI) tends to be more sensitive. In fact, MRI improved prediction of inoperability over CT with 90.6% sensitivity compared to 25% [13,14].

In comparison to surgical PCI, MRI PCI accurately classified tumor volume in 91% of patients, compared to just 50% with CT scanning. Notably, in small-bowel areas (sites 9–12), MRI had 92% precision versus 48% for CT [15].

However, if the radiological PCI is 20 or higher, the chances of optimal cytoreduction are extremely low; a PCI ranging from 0 to 10 may cause a favorable outcome, while several small tumor nodules may be present and result in an unfavorable outcome. A radiological PCI of 10–20 is indicative, but not definitive, of an unfavorable result.

Preoperative MRI and CT of the abdomen and pelvis are essential in evaluating the severity of peritoneal and visceral disease, avoiding unnecessary procedures in patients whose tumors are too extensive and cannot be sufficiently cytoreduced, and can recommend appropriate therapeutic strategies of treatment. Tumor burden influences the efficacy of intraperitoneal therapies.

## 8. Qualitative Assessment of Peritoneal Carcinomatosis: Disease Potentially Non-Resectable Based on Distribution

The second purpose of preoperative imaging is to evaluate anatomic locations of peritoneal disease correlated with the risk of an adverse effect, technically more complicated resections, or suboptimal cytoreduction. The idea of “concerning radiologic features” discovered on preoperative imaging was used to alert the multidisciplinary team to the probability of incomplete cytoreduction. These radiological findings are associated with the involvement of pelvic structures, retroperitoneal structures, gastro-hepatic or hepatoduodenal ligaments with gastric outlet obstruction, and infiltration of the small bowel and its mesentery.

### 8.1. Pelvic Involvement

**Figure 2:**
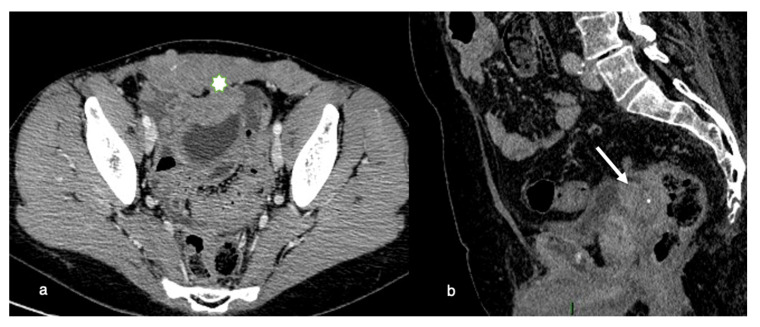
Pelvic involvement. (**a**) Axial contrast-enhanced CT image shows a diffuse and irregular thickening of the anterior pelvic wall (asterisk) caused by the extension of tumoral tissue to adjacent muscles with penetration into the abdominal wall (**b**) Sagittal contrast-enhanced CT image shows a nodular cancerous implant located in the recto-sigmoid pouch (arrow) invading the bladder posterior wall (trigone of the bladder).

Invasive disease in the pelvis has significant implications for optimal resection. If the primary tumor is within 3 mm of the pelvic sidewall or surrounds more than 90% of the diameter of the iliac arteries with a vascular encasement, invasion of the pelvic wall may be assumed. Total cytoreduction can also be hampered by evidence of bladder trigone invasion (Figure 2).

If the seminal vesicles are invaded, they must be resected, with definite implications for sexual function postoperatively.

### 8.2. Retroperitoneal Involvement 

**Figure 3:**
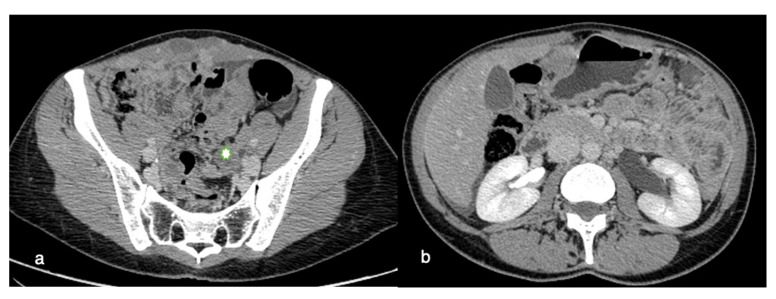
Retroperitoneal involvement. Axial contrast-enhanced CT images show involvement of the left pelvic ureter (**a**) encased by tumoral tissue (asterisk) associated with left hydronephrosis (**b**).

The retroperitoneum is an anatomic site with few cytoreductive surgical options. Ureters are commonly thought to be unresectable anatomic areas for peritoneal metastases. Ureter obstruction is caused by high-grade cancer that spreads around the ureter. A successful resection with margins on the ureter and surrounding cancer is unlikely. Deep infiltration along the fascicles of the psoas muscle can occur and is difficult to remove surgically, potentially requiring a psoas muscle resection (Figure 3).

Not only does mesentery and/or para-aortic lymph node penetration mean a bad outcome, since full cancer resection can be challenging, but it also suggests high-grade invasive disease that is spreading beyond the peritoneal space [15].

### 8.3. Gastrohepatic or Hepatoduodenal Ligaments and Gastric Outlet Obstruction 

**Figure 4:**
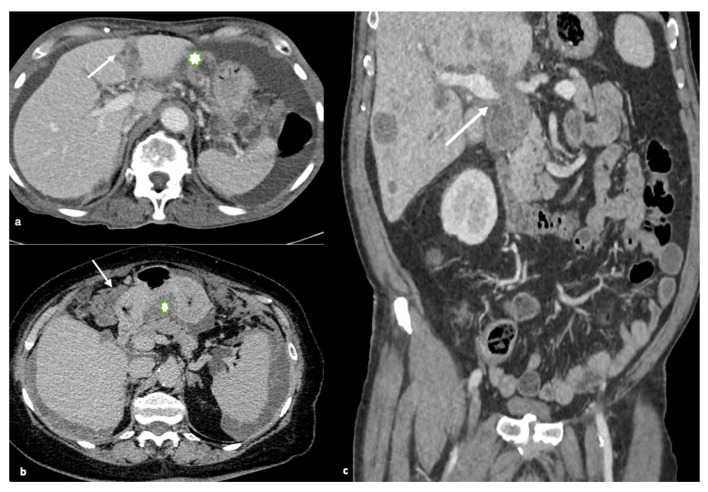
Gastro-hepatic or hepatoduodenal ligaments and gastric outlet obstruction. (**a**) Axial contrast-enhanced image shows multiple peritoneal deposits involving the falciform ligament (arrow) and gastro-hepatic ligament (asterisk), (**b**) Axial contrast-enhanced images show the spread of disease anterior (arrow) and posterior to the stomach (in the lesser sac; asterisk). This latter condition may result in gastric outlet obstruction, (**c**) Coronal contrast-enhanced image demonstrates a large, bulky mass in the porta hepatis and hepatoduodenal ligament with mass effect on the portal vein (arrow).

The falciform ligament, gallbladder fossa, and periportal space must all be closely examined. Carcinomatous lesions in these areas, particularly those larger than 2 cm, are often predictive of non-optimal debulking. High-grade nodules with sclerotic characteristics could limit or even obstruct the normal hepatic ducts. Tumors with large volumes in the gastrohepatic ligament can also cover the left gastric artery. Enhancing soft tissue that extends along the portal veins is direct evidence of tumor spread along this peritoneal pathway, as is the presence of ill-defined infiltration of the gallbladder fossa (Figure 4).

The spread of disease anterior and posterior to the stomach’s antrum can cause gastric outlet obstruction.

### 8.4. Infiltration of the Small Bowel and its Mesentery 

**Figure 5:**
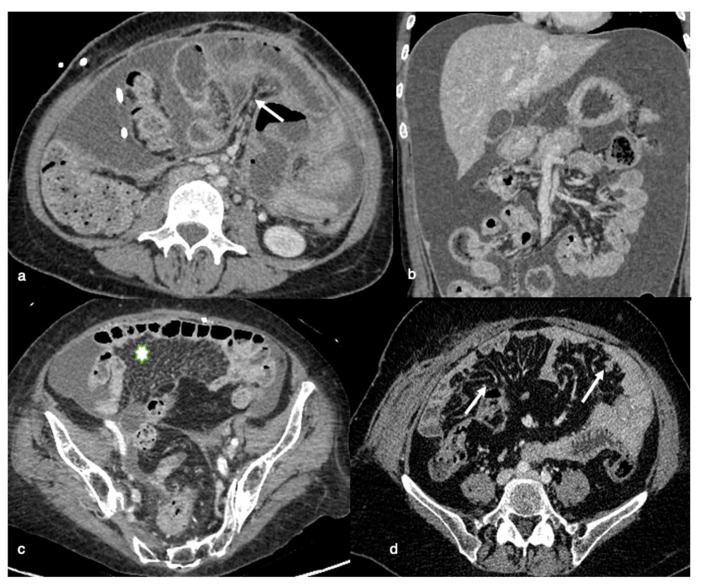
Infiltration of the mesentery. Axial contrast-enhanced image (**a**) shows a diffuse infiltration and thickening of mesenteric reflections (arrow) causing diffuse rigidity and retraction of the mesentery, with contraction of bowel toward the retroperitoneum causing a clumped appearance on the coronal CT image (**b**). Axial contrast-enhanced image shows the “stellate pattern” produced when straightened blood vessels held rigid by the thickened mesentery look like stars in the sky (asterisk in (**c**)); infiltration of the mesentery can also be represented by nodular visceral peritoneal thickening with multiple small nodules in the mesenteric layers (arrows in (**d**)).

Mesenteric disease may have a wide range of appearances in CT imaging, ranging from generalized penetration (misty mesentery) or clustered small ovoid soft-tissue densities, to confluent, massive, irregular soft-tissue masses characteristically scattered across the superior mesenteric vessels, or affecting both the serosa and the adjacent mesentery. Rigidity and retraction are caused by extensive mesenteric interaction, with contraction of the bowel into the retroperitoneum inducing the clumped form (Figure 5).

The presence of bowel loops and the serosa, both known to be distal components of the mesentery, should be quantified (diffuse bowel involvement with partial obstruction at more than one location or substantial involvement of jejunal regions) where their extensive involvement is linked to non-resectable disease [16]. In comparison, mesenteric root infiltration is considered an absolute criterion of non-resectability.

### 8.5. Extraperitoneal Disease Detection

Some anatomic sites are “blind spots” that are unavailable during laparoscopy, and any signs of illness in these sites must be recorded by the radiologist. Only radiologic imaging can visualize small hepatic or splenic parenchymal metastases, central hepatic metastases, intraluminal deposits in the gastrointestinal tract, extra-regional lymph nodes, and pleural metastases, so the imaging should be explained in the radiologist’s report to allow the surgeon to use an effective treatment technique and prepare the surgery [16].

## 9. Relative Contraindications to PIPAC or Laparoscopic Approach

Adhesions, obliteration of the peritoneal space (omental cake or great nodules), organomegaly, intestinal distension, or portal hypertension/cirrhosis have an impact on a procedure’s access to the abdomen.

According to a systematic examination, access to the abdomen is largely dependent on patient selection and surgical ability, and after cytoreductive surgery, peritonectomy, and HIPEC, it shows a non-access rate higher than after other procedures [17].

In particular, in over 30% of patients with a history of previous surgery, the intestine or other organs are directly adhered to the abdominal scar, making blind access to these areas difficult. Peritoneal adhesions may be detectable during a normal computed tomography (CT) or magnetic resonance imaging (MRI) study performed to determine the primary disease phase. Peritoneal adhesion is detected through indirect signs on CT and MRI.

**Figure 6:**
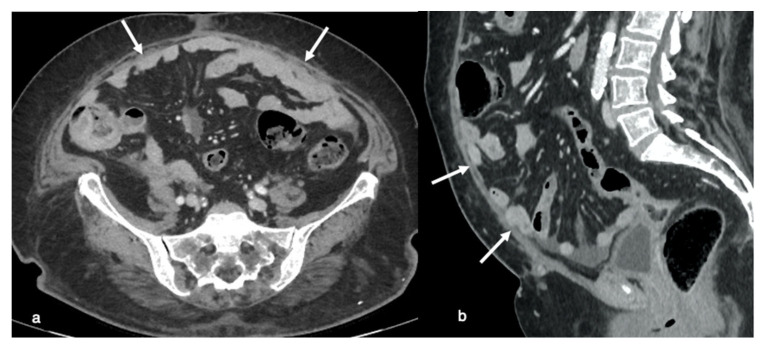
Relative contraindications to PIPAC or laparoscopic approaches. Axial (**a**) and sagittal MPR (**b**) contrast-enhanced CT-images show a focal obscuration of pro-peritoneal fat associated with closely adherent omentum and small bowel loops (arrows), indicative of anterior entero-parietal adhesions.

Imaging results in patients with anterior entero-parietal adhesions at the operative site include focal obscuration of pro-peritoneal fat associated with closely adherent omentum and small bowel loops (Figure 6). Indirect symptoms can also be caused by an extrinsic indentation or kink over a bowel loop, which can induce mucosal fold distortion or luminal restriction. Peritoneal adhesion is also assumed as linear or curvilinear soft tissue stands, extending up to another bowel loop or peritoneal surface [18].

**Figure 7:**
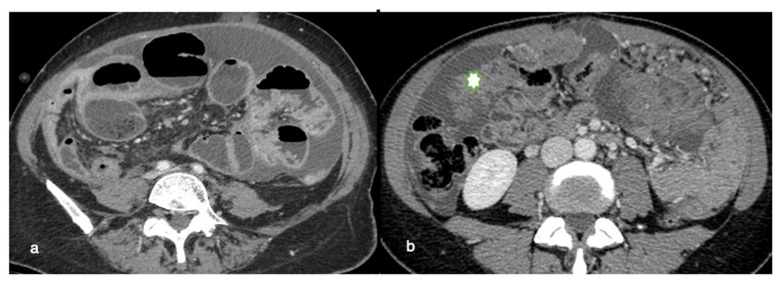
Relative contraindications to PIPAC or laparoscopic approaches. CT axial images can easily detect dilated bowel loops, indicative of intestinal occlusion (**a**) or diffuse omental thickening (“omental cake”; asterisk) (**b**). These conditions may represent a relative contraindication to PIPAC, being responsible for complex access to the abdominal cavity with a consequent higher risk for iatrogenic bowel lesions. The presence of omental masses can also potentially reduce treatment efficacy due to difficulties in drug penetration.

Intestinal (sub)occlusion of dilated bowel loops may also be a relative side effect of the laparoscopic approach since it is associated with a greater chance of access injury or iatrogenic bowel lesions (Figure 7). Laparoscopy is challenging in scenarios in which there are diffusely dilated small bowel loops, since the working space available in the pneumoperitoneum is restricted. Furthermore, small bowel manipulation and retraction in this context are associated with an increased risk of serosal tears or enterotomy [19,20].

Chemical peritonitis caused by PIPAC, along with corresponding “burned-like” tissue damage and small bowel edema, can result in a complete loss of small bowel function, even in sub-occlusive disease, and for this reason should be prevented [21].

PIPAC can also be contraindicated in patients with a stiff and coarse abdominal wall as a symptom of broad tumor load of the visceral and parietal peritoneum, with peritoneal space obliteration attributable to omental cake or great nodules (Figure 7). Access to the abdominal cavity is complicated in these cases, and the chance of bleeding complications from these tumor masses is elevated. Large tumors and bulky deposits have a lower response to PIPAC when compared to miliary patterns, likely because of lower drug penetration [22]. In such cases, tumor-associated adhesions are frequently prominent, increasing the likelihood of iatrogenic bowel lesions.

In cirrhotic patients, the risks associated with abdominal access are often attributed to abdominal wall varices that raise the possibility of bleeding at the point at which the laparoscopy takes place.

## 10. Conclusions

Pressurized intraperitoneal aerosol chemotherapy (PIPAC) is a new technique in the palliative treatment of non-resectable peritoneal metastasis (PM) or recurrences resistant to chemotherapy; it is based on laparoscopically administered, aerosolized chemotherapy into the hyperbaric capnoperitoneum. In comparison to conventional chemotherapy, PIPAC delivers considerably higher concentrations of chemotherapy into the peritoneum while maintaining a low concentration in the systemic circulation, avoiding the harmful effects of systemic administration.

Radiologists are important members of the multidisciplinary teams that treat patients with PC, assisting surgeons in determining the efficacy of the PIPAC technique, allowing accurate treatment preparation, establishing which kind of patients may benefit from CRS, and detecting imaging observations that avoid a laparoscopic solution or suggest higher risks for non-complete cytoreductive surgery. Our radiological checklist is a proposal that could help radiologists better select patients who are good candidates for PIPAC from patients who may not benefit from it. It is necessary to underline that a radiological checklist needs to be discussed in a multidisciplinary board to really obtain a consensus for PIPAC indication in patients with advanced peritoneal carcinomatosis. However, further future studies are needed to confirm our results and obtain external validation of our radiological checklist.

## Data Availability

Data sharing is not applicable to this article as no datasets were generated or analyzed.

## Appendix A

After multidisciplinary discussion, evidence reported in the literature and perceptions based on our internal experience were integrated with the aim of obtaining a radiological checklist. Our proposal needs to be externally validated for confirming our suggestions. However, it could be used to better identify good candidates for PIPAC and those for whom it may be futile, with results that have to be discussed in a multidisciplinary board to really obtain a consensus for PIPAC indication in patients with advanced peritoneal carcinomatosis.

### Appendix A.1. Radiological CHECKLIST for Evaluating Inclusion/Exclusion Criteria for PIPAC in Patients with Advanced Peritoneal Carcinomatosis

#### Appendix A.1.1. Are There Radiological Exclusion Criteria for Surgery?

- Check radiological PCI score (surgically unfavourable outcomes are potentially considered for a PCI of more than 10, depending on primary tumor histology)
- Exclude distribution of disease in potentially unresectable abdominal sites:
  - Check pelvic structures carefully
  - Check retroperitoneum carefully
  - Exclude gastrohepatic and hepatoduodenal ligaments involvement
  - Evaluate mesentery and small bowel in order to exclude eventual tumor infiltration
- Don’t forget extraperitoneal involvement
  - Exclude parenchymal metastases
  - Exclude extra-regional lymph nodes
  - Evaluate thoracic structures (pulmonary/pleural involvement)

#### Appendix A.1.2. Can We Exclude PIPAC? Are There Any Laparoscopic Contraindications?

- Identify eventual abdominal adhesions
- Exclude intestinal (sub)occlusion radiological signs
- Evaluate peritoneal implant size to exclude great peritoneal nodules or diffuse omental thickening
- Check for the presence of eventual abdominal wall varices
